# Supportive Care in Radiotherapy Based on a Mobile App: Prospective Multicenter Survey

**DOI:** 10.2196/10916

**Published:** 2018-08-30

**Authors:** Rami A El Shafie, Dorothea Weber, Nina Bougatf, Tanja Sprave, Dieter Oetzel, Peter E Huber, Jürgen Debus, Nils H Nicolay

**Affiliations:** ^1^ Department of Radiation Oncology Heidelberg University Hospital Heidelberg Germany; ^2^ National Center for Research in Radiation Oncology Heidelberg Institute for Radiation Oncology Heidelberg Germany; ^3^ Institute of Medical Biometry and Informatics Heidelberg University Hospital Heidelberg Germany; ^4^ Department of Radiation Oncology German Cancer Research Center Heidelberg Germany; ^5^ Department of Radiation Oncology University Medical Center Freiburg Freiburg Germany; ^6^ German Cancer Consortium, Partner Site Freiburg Faculty of Medicine University of Freiburg Freiburg Germany

**Keywords:** mHealth, radiotherapy, mobile app, quality of life, surveillance, patient-reported outcome, acceptance, smartphone, mobile phone

## Abstract

**Background:**

Consumer electronics and Web-enabled mobile devices are playing an increasing role in patient care, and their use in the oncologic sector opens up promising possibilities in the fields of supportive cancer care and systematic patient follow-up.

**Objective:**

The objective of our study was to assess the acceptance and possible benefits of a mobile app–based concept for supportive care of cancer patients undergoing radiotherapy.

**Methods:**

In total, 975 patients presenting for radiotherapy due to breast or prostate cancer were screened; of them, 200 owned a smartphone and consented to participate in the survey. Patients were requested to complete a questionnaire at 2 time points: prior to the initiation (T0) and after the completion (T1) of radiotherapy. The questionnaire included questions about the habits of smartphone usage, technical knowledge and abilities of the participants, readiness to use a mobile app within the context of radiotherapy, possible features of the mobile app, and general attitude toward the different aspects of oncologic treatments. For quantitative analysis, sum scores were calculated for all areas of interest, and results were correlated with patient characteristics. Additionally, answers were quantitatively compared between time points T0 and T1.

**Results:**

Median patient age was 57 (range 27-78) years. Of the 200 participants, 131 (66.2%) reported having the ability to use their smartphones with minimal to no help and 75.8% (150/200) had not used their smartphones in a medical context before. However, 73.3% (146/200) and 83.4% (166/200) of patients showed a strong interest in using a mobile app for supportive care during radiotherapy and as part of the clinical follow-up, respectively. Patients most commonly requested functionalities regarding appointment scheduling in the clinic (176/200, 88.0%) and the collection of patient-reported outcome data regarding their illness, therapy, and general well-being (130/200, 65.0%). Age was identified as the most influential factor regarding patient attitude, with patients aged <55 years being significantly more inclined toward and versed in smartphone use (*P*<.001). The acceptance of mobile apps was significantly higher in patients exhibiting a Karnofsky performance index <80% (*P*=.01). Support in the context of therapy-related side effects was judged most important by patients with poor clinical performance (*P*=.006). The overall acceptance of mobile apps in the context of radiotherapy surveillance was high at a median item sum score of 71.4/100 and was not significantly influenced by tumor stage, age, gender, treatment setting, or previous radiotherapies.

**Conclusions:**

The acceptance of mobile apps for the surveillance and follow-up of cancer patients undergoing radiotherapy is high; this high acceptance level will serve as a basis for future clinical trials investigating the clinical benefits of mobile app–based treatment support. Introduction of mobile apps into the clinical routine should be considered as an opportunity to improve and intensify supportive treatment for cancer patients.

## Introduction

The usage of consumer electronics and Web-enabled mobile devices is steadily increasing in the medical sector. Mobile health care apps are summarized by the World Health Organization under the term “mHealth” (mobile health) and have recently shown a significant rise in availability and market share [1,2]. Far from being but a response to the increased availability of smartphones and similar devices, the increased role of mHealth can be interpreted as a reaction to structural and demographic challenges faced by health care providers in today’s society. As patient empowerment and shared decision making become increasingly valued in health care, mHealth can provide the means of incorporating those values into modern treatments, for example, facilitating the collection of patient-reported outcomes or providing information about disease management and prevention [3]. The main arguments that favor mHealth approaches include the possibility to overcome geographic distances and language barriers or selectively address special needs of patient subgroups, such as children or ethnic minorities [4,5].

Mobile apps have been well implemented for the management of highly prevalent conditions such as diabetes, obesity, or cardiovascular diseases [4,6,7]. Cancer, with generally improved long-term survival rates, is developing into a chronic illness with similar requirements such as close patient monitoring and extensive and long-term supportive care [3]. Few mobile apps have been established for supportive cancer care, and the areas of use are still limited [8]. Furthermore, cancer-related mHealth apps and online resources often lack clinical validation. Several reviews examining the clinical benefits of available mobile apps have shown that the overall accuracy, actuality, and systematic validity of the provided information have rarely been confirmed in clinical studies [9-11]. To date, there are no validated mobile apps specifically for the management of patients receiving radiotherapy, a treatment modality with its very own subset of possible side effects and requirements regarding surveillance and supportive care [12].

The acceptance of mobile apps and Web-based medical resources by cancer patients remains largely unclear, especially because patient satisfaction in this context is rarely assessed systematically [11]. This is even more critical as this patient cohort is extremely heterogeneous regarding patient age, technological affinity and skills, income status, and individual burden and distress attributable to this often severe illness.

This prospective study aimed to systematically examine the acceptance of mobile apps by cancer patients undergoing radiotherapy by conducting a systematic survey at the Departments of Radiation Oncology of the National Center for Tumor Diseases, Heidelberg University Hospital and the German Cancer Research Center in Heidelberg. It specifically addresses the patients’ readiness and inclination toward the usage of a mHealth-based approach for additional supportive care in the context of radiotherapy. Furthermore, patient-side infrastructure, such as technical skills, reachability, or mobile data availability, was assessed. The possible functionalities and features of a supportive mobile app were systematically evaluated, and the potential influences of radiotherapy and other predictive and clinical factors on patients’ attitude were investigated.

## Methods

### Patient Characteristics

**Figure 1:**
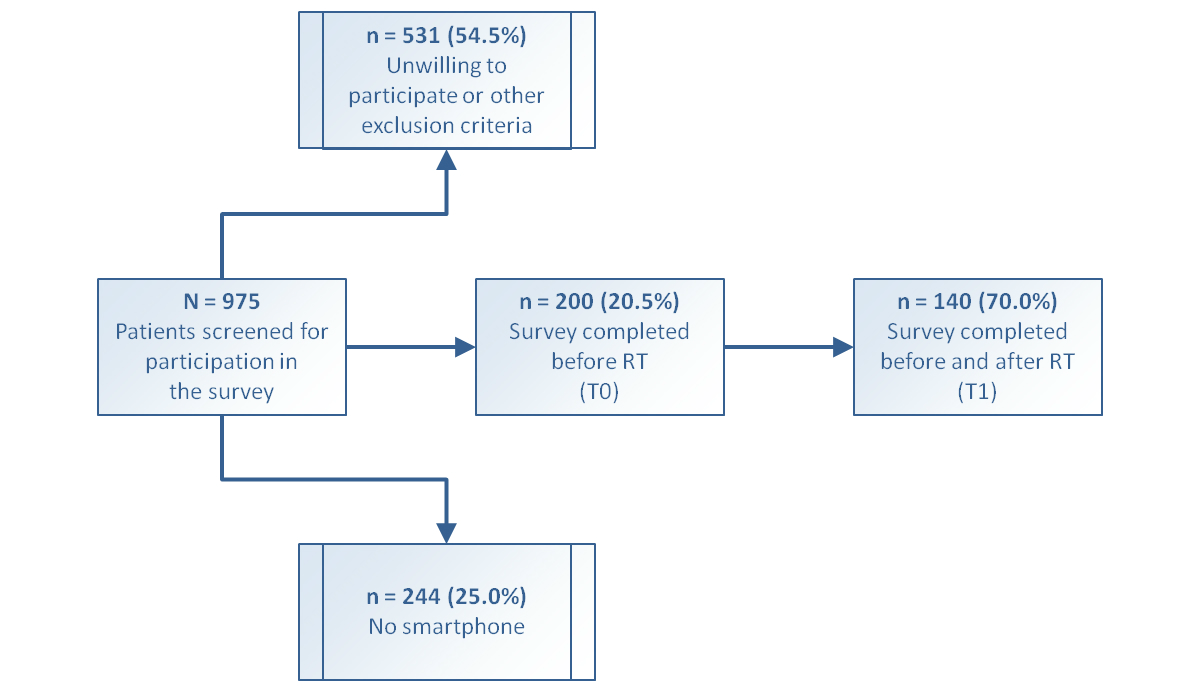
Flowchart illustrating patient screening and recruitment workflow. RT: radiotherapy.

A total of 975 cancer patients presenting for radiotherapy at the above-mentioned institutions for breast or prostate cancer were screened for participation in this survey. Of them, 200 patients owned a smartphone and consented to participate. All the participants were asked to complete a survey prior to the initiation of radiotherapy (T0) and again upon the completion of the treatment (T1). Patient characteristics are presented in Table 1, and a flowchart of the recruitment workflow is illustrated in Figure 1. This prospective survey was approved by the Heidelberg University Independent Ethics Committee on February 15, 2017 (approval #S-007/2017).

### Survey Methods

The survey form consisted of a standardized paper questionnaire containing 27 items (Q1-Q27) about smartphone use. The questionnaire was developed by experienced radiation oncologists with the help of a biomedical informatist and biostatistician and was tested on a small group of patients to allow room for clarifications and corrections before its distribution within this survey. The types of questions included multiple-choice questions, requiring the patients to choose one or several answers out of 2, 4, or 5 provided options. Two were polar questions, requiring the patients to choose either “yes” or “no.” Five questions prompted the patients to additionally fill in optional free text. Items assessed the habits of smartphone usage (Q3, Q7, and Q8), assessed technical knowledge and abilities in smartphone usage (Q4-Q6), assessed readiness to use a smartphone app within the context of cancer and radiotherapy (Q9, Q14, Q20-22, and Q27), suggested features for a potential radiotherapy-related mobile app (Q10-Q13), suggested the timeframe of reachability for smartphone notifications (Q23-Q25), and assessed the general attitude toward the different aspects of radiotherapy (Q15-Q19 and Q26). Additionally, patient- and disease-related information was collected. An English version of the survey questionnaire is provided in Multimedia Appendix 1.

**Table 1:** Patient characteristics before radiotherapy (N=200).

Characteristics		Value
**Age (years)**		
	Mean (SD)	57.2 (11.08)
	Median (range)	57 (27-78)
	Q1-Q3	50-65
**Gender, n (%)**		
	Male	85 (42.5)
	Female	115 (57.5)
**Diagnosis, n (%)**		
	Breast cancer	115 (57.5)
	Prostate cancer	85 (42.5)
**Treatment setting, n (%)**		
	Curative	169 (84.5)
	Palliative	31 (15.5)
**T status, n (%)**		
	is	4 (2.0)
	1	88 (44.0)
	2	65 (32.5)
	3	36 (18.0)
	4	4 (2.0)
	X	1 (0.5)
	Unknown	2 (1.0)
**N status, n (%)**		
	0	132 (66.0)
	+	19 (9.5)
	1	38 (19.0)
	2	4 (2.0)
	3	4 (2.0)
	X	3 (1.5)
**M status, n (%)**		
	0	159 (79.5)
	1	34 (17.0)
	X	7 (3.5)
**Initial Karnofsky performance index, n (%)**		
	60	3 (1.5)
	70	19 (9.5)
	80	43 (21.5)
	90	68 (34.0)
	100	67 (33.5)
**Previous radiotherapy, n (%)**		
	No	144 (72.0)
	Yes	56 (28.0)
**Tumor stage^a^, n (%)**		
	Early	102 (51.0)
	Advanced	98 (49.0)

^a^Early tumor stage: Tis/1/2, N0, M0; advanced tumor stage: T3 or above, N1 or above, M1.

### Statistical Analysis

To allow for quantitative comparison, a simple scoring method was devised in which the aforementioned areas of interest (AOI) were considered as the subscales of the questionnaire. Within each subscale, every question was weighted by the number of possible answers, and the points were divided equally between the provided answers. Questions Q5, Q10, and Q13 were taken out of the score because they only provided qualitative information. The sum for every subscale was calculated and used for the comparison. Detailed information about the scoring system is provided in Multimedia Appendix 2.

For descriptive analyses, continuous variables were presented as mean (SD) and median (IQR and min and max) and categorical variables as absolute and relative frequencies. Group comparisons were made according to tumor stage, age (below and above 55 years), Karnofsky performance scale index (KPI), previous courses of radiotherapy, treatment setting, and gender [13]. Wilcoxon rank sum test for ordinal scaled variables and chi-square test for categorical variables were used to evaluate potential differences between patients in the mentioned groups. Group differences were assessed for all subscales and, additionally, for all questions included in the calculation for one of the subscales. To evaluate the differences at time points T0 and T1, Wilcoxon signed-rank test for paired ordinal scaled data and McNemar test for categorical variables were used. All statistical analyses were performed using R software (version 3.4.3, The R Foundation for Statistical Computing, Vienna, Austria).

## Results

### Descriptive Analysis

Of all patients, 73.2% (146/200) indicated that they were using mobile data on their smartphones, and the common usage included social media apps (45/200, 22.5%), picture taking and Web browsing (86/200, 43.0%), or further apps (48/200, 24.0%); 10.5% (21/200) of the patients used their smartphones for voice calls and instant messaging. Only 24.2% (48/200) of the patients indicated having used their smartphones in a health-related context before; 66.2% (131/200) of the patients stated that they never or rarely required assistance in using their smartphones and 63.9% (124/200) estimated their technical skills in this regard to be solid or advanced.

Patients showed a high overall readiness to use a mobile app in the context of radiotherapy: 73.3% (146/200) of all patients judged using a dedicated mobile app for additional supportive care during their treatment as helpful or very helpful. Mobile apps usage was judged especially helpful in providing support for the occurrences of treatment-associated toxicities (163/200, 81.8%, helpful or very helpful). The favored frequencies at which patients would be willing to answer short app-based queries regarding their well-being or general symptoms were weekly (98/100, 50.8%) and as required (37/200, 19.2%). Thirty-two out of 200 patients (16.6%) wished to do this only at the beginning and end of therapy. Concerns regarding data security were voiced by 12.2% (24/200) of patients. These concerns were somewhat more frequent in patients older than 55 years, although the difference was not significant compared with patients younger than 55 years (13.7% vs 10.0%, *P*=.16).

The most requested feature of a mobile app was assistance in appointment making for radiotherapy and consultations (176/200, 88.0%), followed by general or specific questions regarding patient well-being during radiotherapy (130/200, 65%). Additionally, patients requested the option to receive answers to questions and information material about diagnosis and therapy. Of all patients, 83.4% (166/200) welcomed the idea of continually using the app during follow-up to stay in touch with the treating physicians, describing this option as helpful or very helpful. The same was true for being contacted by the treating physician if medical warning signs were detected (182/200, 91.6%, helpful or very helpful). A reminder feature for scheduled follow-up examinations was favored by 81.5% (163/200) of patients.

Of the 200 patients, 21.8% (43/200) indicated their timeframe for reachability via smartphone between 7 am and 11 pm to be at least 12 hours; 40.1% (79/200) of patients indicated it to be between 2 and 12 hours. Regarding smartphone notifications about missed calls, instant messages, or push notifications, 75.4% (147/200) of patients stated that they would review those within a maximum timeframe of 2 hours or shorter; 21.5% (42/200) of patients answered “within 12 hours,” and only 3.1% (6/200) would need “2 days or longer.” The same was true for app-specific notifications regarding radiotherapy: the percentages were 67.8% (132/200) for 2 hours or less, 26.2% (51/200) for 12 hours, and 6.2% (12/200) for 2 days or longer.

**Figure 2:**
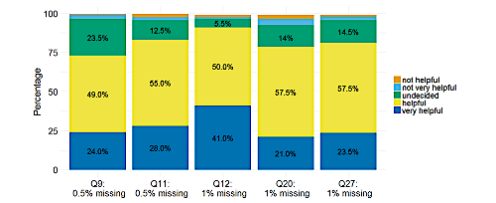
Distribution of the answers provided to selected questions regarding the helpfulness of mobile app–based therapy support in different situations.

**Figure 3:**
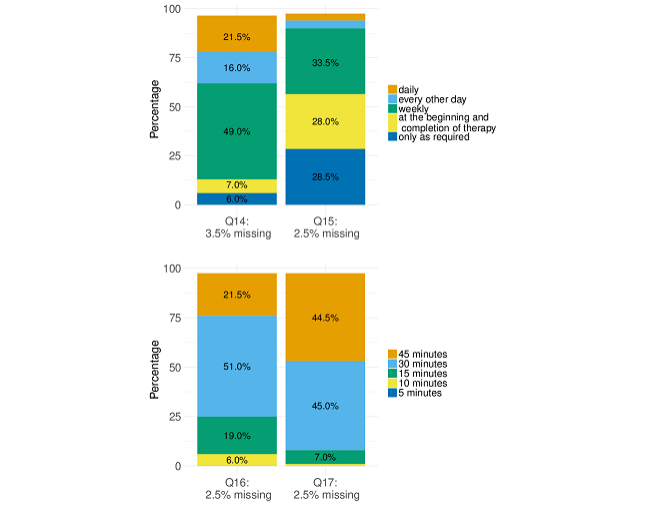
Distribution of the answers provided to selected questions regarding the favored frequencies of consulting a physician (Q14) or answering app-based health-related queries (Q15) as well as maximum acceptable waiting times for daily radiotherapy (Q16) or for a spontaneous medical consultation, if required (Q17).

In addition to the above-mentioned aspects of smartphone and app usage, the survey enquired about the general and organizational aspects of radiotherapy that could be improved using a smartphone app. Regarding the desired frequency of consultations with a supervising physician during radiotherapy, 34.4% (67/200) of patients favored weekly appointments, followed by 29.2% (57/200) favoring appointments “only as required.” Regarding consultations, a waiting period of up to 30 minutes was considered acceptable by 53.4% (106/200) of patients. However, regarding daily radiotherapy, the acceptable waiting period was shorter: 25.7% (50/200) of patients opted for 15 minutes or less and 6.2% (12/200) for even 10 minutes or less. Detailed information about the answers provided to all survey items is illustrated in Figures 2 and 3 and in Multimedia Appendices 3 and 4.

In Figure 2, Q9 corresponds to “Use of a dedicated smartphone app for support during radiotherapy”; Q11 to “Staying in touch via smartphone app during follow-up”; Q12 to “Being contacted about medical warning signs via a smartphone app”; Q20 to “App collecting relevant medical information prior to consultation”; and Q27 to “App-based supportive care in the context of treatment side effects.”

### Areas of Interest

**Figure 4:**
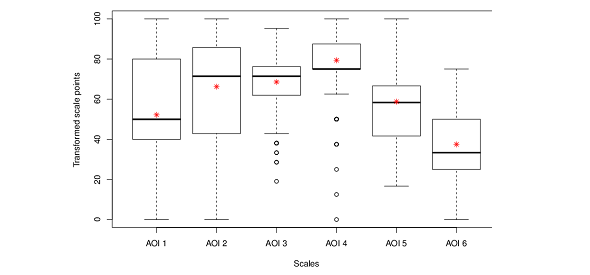
Scores for the different areas of interest (AOI) covered by the questionnaires. The asterisks indicate the mean value of the corresponding AOI.

For quantitative evaluation, the items in the questionnaire were grouped according to subject to calculate the scores for AOI, as described above. The scores were transformed to represent a value between 0 and 100, where a higher score represents a higher inclination or acceptance toward the use of a smartphone app [14-16]. The scores for AOI as calculated from the analysis of all returned questionnaires filled out at T0 are indicated in Table 2 and Figure 4. The highest scores were achieved for AOI 3 (“readiness to use a dedicated app within the context of radiotherapy”) and AOI 4 (“suggested features of a mobile app”), achieving median values of 71.4 (Q1-Q3=61.9-76.2) and 75.0 (Q1-Q3=75.0-87.5), respectively. AOI 3 and 4 translate most directly into a high acceptance for the presented app-based model of therapy support. A similar median value of 71.4 (Q1-Q3=42.9-85.7) was calculated for AOI 2 (“technical knowledge and abilities”). AOI 5 (“timeframe of reachability”) and 6 (“general attitude”) provided mostly qualitative information regarding reachability and the setting and frequency of medical consultations during treatment. The median score of 58.3 (Q1-Q3=41.7-66.7) for AOI 5 translates into an average reachability within 2 hours during a daily timeframe of 2 to 12 hours for the majority of patients. The score of 33.3 (Q1-Q3=25.0-50.0) for AOI 6 shows a general acceptance for waiting periods of up to 30 minutes for a medical consultation.

### Influence of Radiotherapy

**Figure 5:**
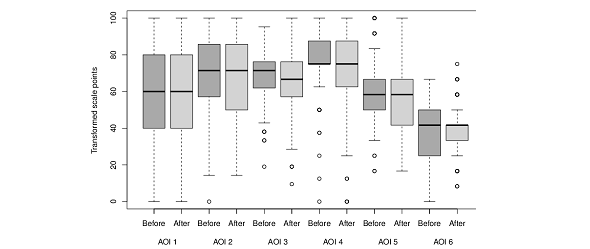
Comparison between the area of interest (AOI) scores of 140 patients who answered the survey at time points T0 and T1.

For 140 patients, survey data before (T0) as well as after the completion (T1) of radiotherapy were available and were compared to evaluate whether having undergone radiotherapy influenced the attitude of patients. A small but statistically significant decrease of 4.7 points in the median transformed score for readiness to use an app within the context of radiotherapy (AOI 3) was detected at T1 (mean 68.6 vs 65.5, *P*<.001, Wilcoxon signed-rank test). The question within AOI 3 leading to this difference addressed the favored frequency of answering symptom-related questions posed by the app (Q14). At T1, patients selected “at therapy start and completion” and “only as required” more frequently instead of “weekly” and “every other day.” The helpfulness of app-based support in the context of toxicity was judged slightly lower at T1 (mean 4.04 before radiotherapy vs 3.88 after radiotherapy, *P*=.03). Regarding the median score for Q12, addressing the helpfulness of being contacted by a physician in case of medical warning signs, the mean/median score was 4.29/4 at T0 and 4.11/4 at T1 (*P*=.03). This question translated into a small but significant difference of 4.4 points in the transformed score for AOI 4 (mean 79.4 before radiotherapy vs 75.0 after radiotherapy, *P*=.04, Wilcoxon signed-rank test). For the other AOIs (AOI 1, 2 and 5, 6), as well as for individual questions, no significant difference was observed between the time points T0 and T1. A comparison of AOI scores between the time points T0 and T1 is illustrated in Figure 5. Detailed information regarding the quantitative comparison between answers provided at T0 and T1 for different survey items is provided in Table 3.

**Table 2:** Scores for the areas of interest (AOI) as calculated from the analysis of all returned questionnaires filled out at T0.

Scale	Description	Items	Mean (SD)	Median	Q1	Q3	Min	Max
AOI 1	Habits of smartphone use	Q3 + Q7 + Q8	52.2 (27.22)	50	40	80	0	100
AOI 2	Technical knowledge and abilities	Q4 + Q6	66.2 (21.39)	71.4	42.9	85.7	0	100
AOI 3	Readiness to use an app	Q9 + Q14 + Q20 + Q21 + Q22+ Q27	68.6 (14.44)	71.4	61.9	76.2	19	95.2
AOI 4	Possible features of a mobile app	Q11 + Q12	79.4 (17.01)	75	75	87.5	0	100
AOI 5	Timeframe of reachability	Q23 + Q24 + Q25	58.8 (18.58)	58.3	41.7	66.7	16.7	100
AOI 6	General attitude	Q15 + Q16 + Q17	37.5 (13.47)	33.3	25	50	0	75

**Table 3:** Quantitative comparison between answers provided at time points T0 and T1 for different survey items for 140 patients who filled out the survey questionnaire twice.

Area of interest (AOI)		*P* value
**AOI 1**		.27
	Q3	.81
	Q7	.94
	Q8	.005
**AOI 2**		.497
	Q4	.98
	Q6	.23
**AOI 3**		.001^a,b^
	Q9	.17
	Q14	<.001^a,b^
	Q20	.23
	Q21	.98
	Q22	<.001
	Q27	.03^b^
**AOI 4**		.04^a^
	Q11	.09
	Q12	.03^a,b^
**AOI5**		.76
	Q23	.68
	Q24	.14
	Q25	.88
**AOI 6**		.21
	Q15	.45
	Q16	.39
	Q17	.13
	Q18	.56
	Q19	.81
	Q26	.77

^a^Lower score after the completion of radiotherapy.

^b^Significant *P* values.

**Table 4:** *P* values for the influence of tumor stage, age, gender, initial Karnofsky performance scale index (KPI), treatment setting (curative vs palliative), and previous radiotherapy on the answers provided to the survey items and area of interest (AOI) scores.

AOI			Tumor stage		Age 55 years		Gender		KPI		Treatment setting		Previous radiotherapy
**AOI 1**			.45		.005^a,b^		.78		.51		.006^a,c^		.26
	Q3	.63		.19		.57		.56		.16		.09	
	Q7	.74		.01^a,b^		.64		.81		.002^a,c^		.48	
	Q8	.047^a,d^		.004^a,b^		.12		.83		>.99		.73	
**AOI 2**			.66		.001^a,b^		.72		.24		.495		.93
	Q4	.73		.003^a,b^		.64		.35		.25		.88	
	Q6	.70		.004^a,b^		.92		.28		.88		.74	
**AOI 3**			.08		.26		.67		.01^a,e^		.32		.89
	Q9	.45		.58		.17		.58		.12		.35	
	Q14	.09		.12		.26		.06		.003^a,f^		.22	
	Q20	.20		.75		.39		.04^a,e^		.94		.77	
	Q21	.29		.16		.67		.15		.91		.84	
	Q22	.67		.88		>.99		.64		.76		.83	
	Q27	.18		.67		.13		.006^a,e^		.63		.99	
**AOI 4**			.09		.86		.203		.12		.92		.90
	Q11	.19		.74		.146		.20		.95		.99	
	Q12	.07		.68		.542		.18		.72		.99	
**AOI 5**			.07		.003^a,b^		.058		.54		.55		.29
	Q23	.94		.06		.106		.30		.77		.19	
	Q24	.07		.002^a,b^		.484		.87		.41		.30	
	Q25	.26		.12		.641		.84		.75		.98	
**AOI 6**			.96		.05		.041^a,g^		.67		.99		.84
	Q15	.71		.009^a,h^		.091		.78		.82		.58	
	Q16	.50		.53		.186		.17		.87		.87	
	Q17	.78		.20		.116		.49		.63		.36	

^a^Significant *P* values.

^b^Higher score for age <55 years.

^c^Higher score for curative treatment setting.

^d^Higher score for advanced tumor stage.

^e^Higher score for KPI <80%.

^f^Higher score for palliative treatment setting.

^g^Higher score for gender (male).

^h^Higher score for age ≥55 years.

### Predictive Factors

To determine possible factors that influenced patients’ attitude toward the usage of a mobile app, we tested several patient characteristics for their impact on survey results. The tested factors were age (<55 years vs ≥55 years), tumor stage (early [defined as tumor stage ≤T2, N0, M0/X] vs advanced), gender, previous radiotherapies, treatment setting (curative vs palliative), and initial KPI (<80% vs ≥80%). Age appeared to have the most sizable impact on the answers provided in the questionnaire, leading to significant differences in usage habits and technical skills as well as reachability. In all cases, younger patients were found to be more inclined toward and versed in more intensive smartphone use (AOI 2, *P*<.001). Interestingly, the favored frequency of seeing a physician during therapy was higher in patients younger than 55 years (*P*=.009). An overview of the survey items significantly impacted by the analyzed patient characteristics is displayed in Table 4.

## Discussion

### Interpretation of Survey Results

We conducted a prospective and systematic survey regarding the habits and skills in smartphone usage, readiness to use a supportive mobile app during and after radiotherapy, and opinions on suggested functionality for such an app among cancer patients undergoing radiotherapy. Our results showed high overall acceptance levels for the usage of mobile technology in the context of radiotherapy, and the proposed functionality and features were considered as helpful by a large majority of patients.

Moreover, our survey showed that the ability to use a dedicated mobile app for additional supportive care (eg, mobile data on smartphone, reachability, and technical skills) on the patient side were present in almost three quarters of the survey population, and the obtained numbers were consistent across the different subareas of this survey. These results suggest a promising potential for an mHealth approach in the field of radiotherapy, although they also outline the need for careful patient selection to avoid the unnecessary burdening of subgroups that are either not willing or not capable and, thus, will likely not benefit. As the ownership of a smartphone and informed consent were prerequisites to participating in the survey, 975 patients were screened, and a total of 200 patients participated. The possible selection bias introduced by this approach has to be considered when generalizing the survey results to all patients undergoing radiotherapy. On the other hand, the results can be valuable in identifying and describing patient subgroups that are most likely to benefit from additional mobile app–based support.

The quantitative analysis of the survey data allowed us to identify age as the most influential factor for patient willingness with younger patients being considerably more inclined toward the use of mobile technology in the context of radiotherapy. Oncological diagnoses are typically associated with certain age groups (eg, prostate cancer, lung cancer, and different subgroups of head and neck cancer). Thus, a degree of patient selection based on age and age-associated diagnosis seams feasible when offering mHealth-based support. On the other hand, prospective clinical evidence exists, showing that patients with unfavorable characteristics regarding age and diagnosis, such as lung cancer patients, can also benefit. This especially holds true if the design of the electronic health (eHealth) or mHealth app integrates the patient’s next of kin to assist in the usage [17,18].

Several studies have shown that patient compliance plays a special role in the successful implementation of mHealth initiatives [19]. Clinical experience in oncology shows patient compliance to be worse in subgroups with lifestyle-related risk factors (eg, heavy smoking and alcohol abuse), potentially making such patients less eligible for mHealth-based support [20,21]. However, evidence exists that offering mHealth support in addition to close clinical surveillance may improve patient compliance, particularly in the abovementioned subgroups, by providing regular prompts and reminders and facilitating adherence to prescribed exercises or supportive regimens [22-24].

The perceived needs of cancer patients for supportive measures are manifold; they may strongly vary depending on culture, diagnosis, prognosis, and associated symptoms and may, hence, influence the acceptance of such measures [25-27]. Our survey showed that, disregarding few minor points, the acceptance of the proposed mobile app approach was high among patients, irrespective of tumor stage, treatment setting, potential previous radiotherapies, and initial clinical performance. Regarding toxicity-related surveillance, acceptance was significantly higher in patients with a reduced performance status. Nevertheless, as poor-performing patients require a different form of intensified personal care, the use of a mobile app alone may show limitations, particularly in the palliative setting [28].

It can be argued that by limiting the present survey to include only prostate and breast cancer patients, a selection bias in favor of patients with favorable prognosis and good clinical performance is introduced, and the generalizability of the results for other cancer patients undergoing radiotherapy could be limited. This potential limitation was accepted to achieve a more homogeneous dataset and ensure timely and systematic survey completion and analysis. However, it should be noted that we included 49.0% of patients with advanced tumor stages, 17.0% of patients with a metastatic disease, and 28.0% of patients who had undergone previous radiotherapy. These patient subgroups feature a different clinical profile characterized by unfavorable prognosis, usually a rapid decline in clinical performance, and a high need for supportive care [29]. The described clinical profile is shared by a majority of patients undergoing radiotherapy for different diagnoses, and the survey results regarding the acceptance of mobile app–-based support did not differ significantly in this subgroup [30-32]. Furthermore, it is of interest that irrespective of age, no further diagnosis-specific influencing factors regarding mobile app acceptance were identified for either breast or prostate cancer patients. This finding, in turn, supports the approach of cautiously extrapolating our results to patients with differing diagnoses.

The helpfulness of app-based supportive care in the context of monitoring potential radiotherapy-induced side effects was judged minimally higher by patients before the beginning of therapy, as was the favored frequency of interaction with the app. These results highlight the importance of providing sufficient information and support before and during the early stages of radiotherapy to address potential fears and worries. Such fears and worries and, thus, the need for additional support are less likely to be observed when the therapy is successfully completed, and the results of our survey accurately mirror this constellation.

### Review of the Literature

The management of cancer patients is an area of special interest for the development of new eHealth and mHealth initiatives because they show promising potential, particularly in the context of supportive care and follow-up [7].

Only one other survey focusing on the acceptance of a dedicated mobile app among cancer patients has been published [33]. Overall results showed convincing similarity in both surveys. However, good or very good technical skills and the willingness to send data to the treating clinic via an app were slightly less frequent in the previous survey than in our survey (54.1% vs 63.9% and 48.5% vs 71.1%, respectively). Moreover, younger patients were generally more willing to use mobile technology in a disease-related context. In comparison, our survey described a more homogeneous and precisely selected cohort, focusing exclusively on patients receiving radiotherapy. Consequently, the specific requirements and circumstances related to this course of treatment are more comprehensively examined.

Ruland and colleagues have reported results similar to those of our survey, testing the usefulness and acceptance of a Web-based, multicomponent eHealth app with special focus on patient self-management among breast and prostate cancer patients [34]. The tested app did not focus exclusively on radiotherapy, and it provided supportive care in a more general manner, featuring message boards and general information sections, among other functionalities. Active usage among the regarded cohort was 64%, which is close to the results of our survey, and in this context, age and diagnosis were reported to significantly impact patient usage [34].

The value of an eHealth or mHealth resource is particularly high when tailored to fit the needs of the target patient cohort because patients are more likely to appreciate the immediately relevant and personalized support [34]. Depending on the diagnosis and therapy regimen, the wide heterogeneity among cancer patients and the very specific needs of different subgroups make them a challenging collective to address as a whole. Consequently, existing mHealth projects have typically been addressing either a specific question or a specific subgroup of cancer patients.

A recent randomized controlled trial by Denis and colleagues has shown a significant median overall survival benefit of 5 months for lung cancer patients who were systematically telemonitored using a mobile app based on patient-reported data and using the dynamics of patients’ clinical symptoms for risk stratification and individualized follow-up [18,35]. Similar approaches have successfully been used in the management of toxicities related to head and neck cancer treatment: computerized screening could facilitate the identification of treatment-related toxicities, and telepractice apps or videoconferencing could assist in the delivery of intensive home-based dysphagia therapy [24,36]. Regarding general supportive care, considerable advances have been made in the development of mHealth interventions to address the common issue of fatigue among cancer survivors. A recent meta-analysis identified 9 completed eHealth studies that revealed a significant beneficial effect of eHealth interventions on fatigued patients with improvements in health-related quality of life and depression [37].

Patients undergoing radiotherapy represent a distinct subgroup of cancer patients with special requirements in terms of supportive care. The nature of radiotherapy results in a specific set of therapy-related side effects and medical issues that require surveillance and potential support. Of the symptoms, the most common are dermatitis, nausea, fatigue, and localized toxicities within the respective treatment region [12]. Furthermore, depending on the diagnosis, patients undergoing radiotherapy vary in terms of age and characteristic profiles regarding individual risk constellations and comorbidities. Based on the data reported here, the usefulness and clinical implementation of a mobile app will be evaluated in a prospective trial (OPTIMISE-1; ClinicalTrials.gov identifier #NCT03168048) for which the dedicated mobile app has been designed according to the requirements of the patients who were assessed [38].

### Conclusion

To the best of our knowledge, this study is the first to prospectively evaluate and demonstrate a high acceptance of and distinct patient requirements for the use of a supportive mobile app in a large homogeneous cohort of cancer patients undergoing radiotherapy. The reported patient acceptance will serve as a basis for future clinical trials that prospectively investigate the benefits of mobile app–based treatment support in routine clinical settings. The introduction of mobile apps into the clinical routine should be regarded as an opportunity to improve and intensify supportive treatment for cancer patients.

## Appendix

Multimedia Appendix 1 Survey questionnaire.

Multimedia Appendix 2 Scoring system.

Multimedia Appendix 3 Descriptive statistics before radiotherapy.

Multimedia Appendix 4 Descriptive statistics after radiotherapy.

## Abbreviations

AOI: area of interest
eHealth: electronic health
KPI: Karnofsky performance scale index
mHealth: mobile health
