# Supplementary material for: Supportive Care in Radiotherapy Based on a Mobile App: Prospective Multicenter Survey
Source: JMIR Mhealth Uhealth. 2018 Aug 30;6(8):e10916. doi: 10.2196/10916 (PMC6137282; doi:10.2196/10916)
Supplement: Multimedia Appendix 1 [file mhealth_v6i8e10916_app1.pdf]

Supplementary file – Survey Questionnaire (English)

1. How old are you? ____________ years

2. What is the name of the manufacturer/operating system of your smartphone?

   A ☐ iPhone (apple) B ☐ Android (e.g. Samsung, htc) C ☐ Windows
   D ☐ other E ☐ unknown

3. Do you use a mobile internet connection (mobile data) on your smartphone or only a wifi-connection?

   A ☐ mobile data B ☐ wifi only

4. Do you need assistance in using your smartphone?

   A ☐ never B ☐ rarely C ☐ sometimes D ☐ frequently E ☐ always

5. Who assists you in using your smartphone?

   A ☐ children/grandchildren B ☐ partner/family C ☐ friends/acquaintances
   D ☐ professional personnel E ☐ others (please specify) ____________________

6. How would you describe your skills in using your smartphone?

   A ☐ minimal B ☐ basic C ☐ solid D ☐ advanced

7. Please categorize your usage habits regarding your smartphone:

   A ☐ voice calls, instant messaging, whatsapp
   B ☐ … additionally taking pictures, web browsing
   C ☐ … additionally social media, facebook
   D ☐ … intensive app use, more than 10 apps downloaded and installed

8. Have you ever used your smartphone for health-related reasons?

   A ☐ yes B ☐ no
   Please specify:____

9. How helpful would you regard the use of a security-certified smartphone app for additional support during radiotherapy?

   A ☐ very helpful B ☐ helpful C ☐ undecided
   D ☐ wenig hilfreich E ☐ nicht hilfreich

10. Please indicate useful features and functionality of such an application from your point of view (multiple selections allowed):

    A ☐ appointment-making for radiotherapy and medical consultations
    B ☐ general queries regarding well-being
    C ☐ specific queries regarding well-being and symptoms in the context of radiotherapy and the illness being treated
    D ☐ other: ____

11. Would you regard it as helpful to use such an application after therapy completion (during follow-up) to stay in touch with your treating physicians?

A ☐ very helpful B ☐ helpful C ☐ undecided
D ☐ not very helpful E ☐ not helpful

12. Would you regard it as helpful to be contacted by a physician if medical warning signs were detected?

A ☐ very helpful B ☐ helpful C ☐ undecided
D ☐ not very helpful E ☐ not helpful

13. Please indicate useful features and functionality of such an application after therapy completion (during follow-up) from your point of view (multiple selections allowed):

A ☐ appointment-making for medical consultations
B ☐ general queries regarding well-being
C ☐ specific queries regarding well-being and symptoms in the context of radiotherapy and the illness being treated
D ☐ reminder for upcoming exams and visits
E ☐ other: ____

14. What interval would you favor for answering short application-based queries regarding your well-being and possible symptoms?

A ☐ daily B ☐ every other day C ☐ weekly
D ☐ at the beginning and completion of therapy E ☐ only as require

15. What interval would you favor for consulting a physician during radiotherapy?

A ☐ daily B ☐ every other day C ☐ weekly
D ☐ at the beginning and completion of therapy E ☐ only as required

16. Please indicate the maximum acceptable waiting period for receiving daily radiotherapy.

A ☐ 5 minutes B ☐ 10 minutes C ☐ 15 minutes
D ☐ 30 minutes E ☐ 45 minutes

17. Spontaneous consultations with a physician pose an organizational challenge and are thus associated with waiting time. Please indicate the maximum acceptable waiting period for such a spontaneous consultation.

A ☐ 5 minutes B ☐ 10 minutes C ☐ 15 minutes
D ☐ 30 minutes E ☐ 45 minutes

18. Do you generally have the feeling to be understood by medical personnel regarding your requests and concerns?

A ☐ always B ☐ mostly C ☐ sometimes
D ☐ rarely E ☐ never

19. Does medical personnel generally take enough time to inquire about all aspects you feel are relevant to your requests and concerns?

    A ☐ always B ☐ mostly C ☐ sometimes
    D ☐ rarely E ☐ never

20. Would you regard it as helpful to answer a short query regarding relevant symptoms and information prior to consulting a physician and have this information presented to the physician to help preparing for your consultation?

    A ☐ very helpful B ☐ helpful C ☐ undecided
    D ☐ not very helpful E ☐ not helpful

21. Would you have reservations about the safety of your medical information when using a security-certified smartphone application?

    A ☐ no reservations B ☐ some reservations C ☐ undecided
    D ☐ substantial reservations E ☐ major reservations

22. Would you agree to having your medical data that is collected during and after therapy anonymized and utilized for medical research?

    A ☐ yes B ☐ no

23. Please indicate the maximum timeframe it would take you to receive and read appointment notifications (for radiotherapy, consultations) on your smartphone (time passed from receiving to reading notification).

    A ☐ 30 minutes B ☐ 60 minutes C ☐ 2 hours
    D ☐ 12 hours E ☐ 2 days or longer

24. In what intervals do you generally check your smartphone for notifications (SMS, missed calls, push notifications)?

    A ☐ 30 minutes B ☐ 60 minutes C ☐ 2 hours
    D ☐ 12 hours E ☐ 2 days or longer

25. During which portion of the daily period between 7am and 11pm would you be reachable by telephone?

    A ☐ < 30 minutes B ☐ < 60 minutes C ☐ < 2 hours
    D ☐ < 12 hours E ☐ more than 12 hours

26. How worried are you about the possibility of side effects occurring during radiotherapy?

    A ☐ not worried B ☐ some worries C ☐ undecided
    D ☐ considerably worried E ☐ extremely worried

27. Would you consider it helpful to receive close-knit regular therapy support via a smartphone application in the context of possible side effects?

    A ☐ very helpful B ☐ helpful C ☐ undecided
    D ☐ not very helpful E ☐ not helpful
