# Supplementary material for: Supportive Care in Radiotherapy Based on a Mobile App: Prospective Multicenter Survey
Source: JMIR Mhealth Uhealth. 2018 Aug 30;6(8):e10916. doi: 10.2196/10916 (PMC6137282; doi:10.2196/10916)
Supplement: Multimedia Appendix 2 [file mhealth_v6i8e10916_app2.pdf]

Supplementary file: Scoring System

Scoring of questions

Scale	Question	Answer	Value
AOI1	Q3	A	2
		B	1
	Q7	A	1
		B	2
		C	3
		D	4
	Q8	A	2
		B	1
AOI2	Q4	A	5
		B	4
		C	3
		D	2
		E	1
	Q6	A	1
		B	2
		C	3
		D	4
AOI3	Q9	A	5
		B	4
		C	3
		D	2
		E	1
	Q14	A	5
		B	4
		C	3
		D	2
		E	1
	Q20	A	5
		B	4
		C	3
		D	2
		E	1
	Q21	A	5
		B	4
		C	3
		D	2
		E	1
	Q22	A	2
		B	1
	Q27	A	5
		B	4
		C	3
		D	2
		E	1
AOI4	Q11	A	5
		B	4
		C	3
		D	2

		E	1
	Q12	A	5
		B	4
		C	3
		D	2
		E	1
AOI5	Q23	A	5
		B	4
		C	3
		D	2
		E	1
	Q24	A	5
		B	4
		C	3
		D	2
		E	1
	Q25	A	1
		B	2
		C	3
		D	4
		E	5
AOI6	Q15	A	1
		B	2
		C	3
		D	4
		E	5
	Q16	A	5
		B	4
		C	3
		D	2
		E	1
	Q17	A	5
		B	4
		C	3
		D	2
		E	1

Scale	Description	Items	Min	Max	Range
AOI1	Habits of smartphoe use	Q3 + Q7 + Q8	3	8	5
AOI2	Technical knowledge and abilities	Q4 + Q6	2	9	7
AOI3	Readiness to use app	Q9 + Q14 + Q20 + Q21 + Q22+ Q27	6	27	21
AOI4	Possible features of mobile app	Q11 + Q12	2	10	8
AOI5	Timeframe of reachability	Q23 + Q24 + Q25	3	15	12
AOI6	General attitute	Q15 + Q16 + Q17	3	15	12

Calculation of sum scores

Calculation of tranformed scale values:

$$\frac{[\mathit{real\ value} - \mathit{smallest\ possible\ value}]}{\mathit{possible\ range}} * 100$$

The smallest possible value of the scales is listed in the column “min” and the possible range is listed in the column “range”. The transformed scale has a minimum value of 0 and a maximum value of 100.
