# Supplementary material for: Supportive Care in Radiotherapy Based on a Mobile App: Prospective Multicenter Survey
Source: JMIR Mhealth Uhealth. 2018 Aug 30;6(8):e10916. doi: 10.2196/10916 (PMC6137282; doi:10.2196/10916)
Supplement: Multimedia Appendix 3 [file mhealth_v6i8e10916_app3.pdf]

**Descriptive statistics before RT**	**Total (n = 200)**
Q3	
1 (B)	52 (26.8%)
2 (A)	142 (73.2%)
Missing	6
Q4	
1 (E)	1 (0.5%)
2 (D)	10 (5.1%)
3 (C)	56 (28.3%)
4 (B)	72 (36.4%)
5 (A)	59 (29.8%)
Missing	2
Q5 (multiple responses possible)	
A	67 (44.4%)
B	62 (41.1%)
C	44 (29.1%)
D	3 (2.0%)
E	1 (0.6%)
Missing	49
Q6	
1 (A)	13 (6.7%)
2 (B)	57 (29.4%)
3 (C)	97 (50.0%)
4 (D)	27 (13.9%)
Missing	6
Q7	
1 (A)	21 (10.5%)
2 (B)	86 (43.0%)
3 (C)	45 (22.5%)
4 (D)	48 (24.0%)
Missing	0

**Descriptive statistics before RT**	**Total**
	**(n = 200)**
Q8	
1 (B)	150 (75.8%)
2 (A)	48 (24.2%)
Missing	2
Q9	
1 (E)	1 (0.5%)
2 (D)	5 (2.5%)
3 (C)	47 (23.6%)
4 (B)	98 (49.2%)
5 (A)	48 (24.1%)
Missing	1
Q10 (multiple responses possible)	
A	176 (88.4%)
B	134 (67.3%)
C	130 (65.3%)
D	12 (6.0%)
Missing	1
Q11	
1 (E)	4 (2.0%)
2 (D)	4 (2.0%)
3 (C)	25 (12.6%)
4 (B)	110 (55.3%)
5 (A)	56 (28.1%)
Missing	1
Q12	
1 (E)	3 (1.5%)
2 (D)	2 (1.0%)
3 (C)	11 (5.6%)
4 (B)	100 (50.5%)
5 (A)	82 (41.4%)
Missing	2

**Descriptive statistics before RT**	**Total (n = 200)**
Q13 (multiple responses possible)	
A	176 (88.0%)
B	130 (65.0%)
C	138 (69.0%)
D	163 (81.5%)
E	7 (3.5%)
Missing	0
Q14	
1 (E)	37 (19.2%)
2 (D)	32 (16.6%)
3 (C)	98 (50.8%)
4 (B)	14 (7.3%)
5 (A)	12 (6.2%)
Missing	7
Q15	
1 (A)	7 (3.6%)
2 (B)	8 (4.1%)
3 (C)	67 (34.4%)
4 (D)	56 (28.7%)
5 (E)	57 (29.2%)
Missing	5
Q16	
1 (E)	43 (22.1%)
2 (D)	102 (52.3%)
3 (C)	38 (19.5%)
4 (B)	12 (6.2%)
Missing	5
Q17	
1 (E)	89 (45.6%)
2 (D)	90 (46.2%)
3 (C)	14 (7.2%)

**Descriptive statistics before RT**	**Total (n = 200)**
4 (B)	2 (1.0%)
Missing	5
Q18	
A	47 (24.1%)
B	137 (70.3%)
C	10 (5.1%)
D	1 (0.5%)
E	0 (0.0%)
Missing	5
Q19	
A	75 (40.0%)
B	105 (55.9%)
C	8 (4.3%)
D	0 (0.0%)
E	0 (0.0%)
Missing	12
Q20	
1 (E)	5 (2.5%)
2 (D)	8 (4.0%)
3 (C)	28 (14.1%)
4 (B)	115 (58.1%)
5 (A)	42 (21.2%)
Missing	2
Q21	
1 (E)	4 (2.0%)
2 (D)	20 (10.2%)
3 (C)	33 (16.8%)
4 (B)	80 (40.6%)
5 (A)	60 (30.5%)
Missing	3
Q22	

**Descriptive statistics before RT**	**Total (n = 200)**
1 (B)	8 (4.1%)
2 (A)	186 (95.9%)
Missing	6
Q23	
1 (E)	12 (6.2%)
2 (D)	51 (26.2%)
3 (C)	59 (30.3%)
4 (B)	44 (22.6%)
5 (A)	29 (14.9%)
Missing	5
Q24	
1 (E)	6 (3.1%)
2 (D)	42 (21.5%)
3 (C)	60 (30.8%)
4 (B)	57 (29.2%)
5 (A)	30 (15.4%)
Missing	5
Q25	
1 (A)	13 (6.6%)
2 (B)	23 (11.7%)
3 (C)	39 (19.8%)
4 (D)	79 (40.1%)
5 (E)	43 (21.8%)
Missing	3
Q26	
A	8 (4.1%)
B	65 (33.0%)
C	60 (30.5%)
D	54 (27.4%)
E	10 (5.1%)
Missing	3
Q27	

**Descriptive statistics before RT**	**Total (n = 200)**
1 (E)	2 (1.0%)
2 (D)	5 (2.5%)
3 (C)	29 (14.6%)
4 (B)	115 (58.1%)
5 (A)	47 (23.7%)
Missing	2
AOI1	
N	192
Missing	8
Mean	52.2
SD	27.22
Median	50
Q1 -- Q3	40 -- 80
Min. -- Max.	0.0 -- 100.0
AOI2	
N	193
Missing	7
Mean	66.2
SD	21.39
Median	71.4
Q1 -- Q3	42.9 -- 85.7
Min. -- Max.	0.0 -- 100.0
AOI3	
N	188
Missing	12
Mean	68.6
SD	14.44
Median	71.4
Q1 -- Q3	61.9 -- 76.2
Min. -- Max.	19.0 -- 95.2
AOI4	
N	197
Missing	3

**Descriptive statistics before RT**	**Total (n = 200)**
Mean	79.4
SD	17.01
Median	75
Q1 -- Q3	75 -- 87.5
Min. -- Max.	0.0 -- 100.0
AOI5	
N	192
Missing	8
Mean	58.8
SD	18.58
Median	58.3
Q1 -- Q3	41.7 -- 66.7
Min. -- Max.	16.7 -- 100.0
AOI6	
N	189
Missing	11
Mean	37.5
SD	13.47
Median	33.3
Q1 -- Q3	25 -- 50
Min. -- Max.	0.0 -- 75.0
