# Supplementary material for: Supportive Care in Radiotherapy Based on a Mobile App: Prospective Multicenter Survey
Source: JMIR Mhealth Uhealth. 2018 Aug 30;6(8):e10916. doi: 10.2196/10916 (PMC6137282; doi:10.2196/10916)
Supplement: Multimedia Appendix 4 [file mhealth_v6i8e10916_app4.pdf]

**Descriptive statistics after RT**	**Total**<br>**(n = 140)**
Q3	
1 (B)	36 (25.9%)
2 (A)	103 (74.1%)
Missing	1
Q4	
2 (D)	4 (2.9%)
3 (C)	40 (28.8%)
4 (B)	54 (38.8%)
5 (A)	41 (29.5%)
Missing	1
Q5 (multiple responses possible)	
A	50 (46.3%)
B	41 (38.0%)
C	27 (25.0%)
D	4 (3.7%)
E	4 (3.7%)
Missing	42
Q6	
1 (A)	6 (4.3%)
2 (B)	39 (27.9%)
3 (C)	66 (47.1%)
4 (D)	29 (20.7%)
Missing	0
Q7	
1 (A)	16 (11.4%)
2 (B)	53 (37.9%)
3 (C)	33 (23.6%)
4 (D)	38 (27.1%)
Missing	0

**Descriptive statistics after RT**	**Total (n = 140)**
Q8	
1 (B)	84 (60.4%)
2 (A)	55 (39.6%)
Missing	1
Q9	
1 (E)	6 (4.3%)
2 (D)	3 (2.2%)
3 (C)	32 (23.0%)
4 (B)	63 (45.3%)
5 (A)	35 (25.2%)
Missing	1
Q10 (multiple responses possible)	
A	116 (85.3%)
B	86 (63.2%)
C	76 (55.9%)
D	9 (6.6%)
Missing	4
Q11	
1 (E)	7 (5.0%)
2 (D)	5 (3.6%)
3 (C)	20 (14.3%)
4 (B)	72 (51.4%)
5 (A)	36 (25.7%)
Missing	0
Q12	
1 (E)	6 (4.3%)
2 (D)	3 (2.1%)
3 (C)	13 (9.3%)
4 (B)	66 (47.1%)
5 (A)	52 (37.1%)
Missing	0

**Descriptive statistics after RT**	**Total (n = 140)**
Q13 (multiple responses possible)	
A	107 (78.7%)
B	79 (58.1%)
C	91 (66.9%)
D	103 (75.7%)
E	5 (3.7%)
Missing	0
Q14	
1 (E)	41 (30.4%)
2 (D)	29 (21.5%)
3 (C)	56 (41.5%)
4 (B)	7 (5.2%)
5 (A)	2 (1.5%)
Missing	5
Q15	
2 (B)	3 (2.2%)
3 (C)	51 (37.2%)
4 (D)	35 (25.5%)
5 (E)	48 (35.0%)
Missing	3
Q16	
1 (E)	42 (30.7%)
2 (D)	57 (41.6%)
3 (C)	34 (24.8%)
4 (B)	3 (2.2%)
5 (A)	1 (0.7%)
Missing	3
Q17	
1 (E)	55 (40.1%)
2 (D)	65 (47.4%)

**Descriptive statistics after RT**	**Total**
	**(n = 140)**
3 (C)	15 (10.9%)
4 (B)	1 (0.7%)
5 (A)	1 (0.7%)
Missing	3
Q18	
A	43 (31.6%)
B	82 (60.3%)
C	10 (7.4%)
D	0 (0%)
E	1 (0.7%)
Missing	4
Q19	
A	66 (48.2%)
B	61 (44.5%)
C	8 (5.8%)
D	1 (0.7%)
E	1 (0.7%)
Missing	3
Q20	
1 (E)	3 (2.2%)
2 (D)	9 (6.6%)
3 (C)	24 (17.5%)
4 (B)	69 (50.4%)
5 (A)	32 (23.4%)
Missing	3
Q21	
1 (E)	4 (2.9%)
2 (D)	14 (10.0%)
3 (C)	23 (16.4%)
4 (B)	58 (41.4%)
5 (A)	41 (29.3%)
Missing	0

**Descriptive statistics after RT**	**Total (n = 140)**
Q22	
1 (B)	5 (3.6%)
2 (A)	133 (96.4%)
Missing	2
Q23	
1 (E)	12 (8.7%)
2 (D)	25 (18.1%)
3 (C)	49 (35.5%)
4 (B)	31 (22.5%)
5 (A)	21 (15.2%)
Missing	2
Q24	
1 (E)	6 (4.4%)
2 (D)	25 (18.2%)
3 (C)	52 (38.0%)
4 (B)	35 (25.5%)
5 (A)	19 (13.9%)
Missing	3
Q25	
1 (A)	10 (7.3%)
2 (B)	14 (10.2%)
3 (C)	25 (18.2%)
4 (D)	50 (36.5%)
5 (E)	38 (27.7%)
Missing	3
Q26	
A	10 (7.2%)
B	51 (36.7%)
C	37 (26.6%)
D	37 (26.6%)
E	4 (2.9%)
Missing	1

**Descriptive statistics after RT**	**Total**
	**(n = 140)**
Q27	
1 (E)	3 (2.2%)
2 (D)	7 (5.1%)
3 (C)	22 (15.9%)
4 (B)	78 (56.5%)
5 (A)	28 (20.3%)
Missing	2
AOI1	
N	138
Missing	2
Mean	56.4
SD	28.72
Median	60
Q1 -- Q3	40 -- 80
Min. -- Max.	0.0 -- 100.0
AOI2	
N	139
Missing	1
Mean	68.4
SD	20.46
Median	71.4
Q1 -- Q3	50 -- 85.7
Min. -- Max.	14.3 -- 100.0
AOI3	
N	133
Missing	7
Mean	65.5
SD	15.86
Median	66.7
Q1 -- Q3	57.1 -- 76.2
Min. -- Max.	9.5 -- 100.0
AOI4	

Descriptive statistics after RT

	Total (n = 140)
N	140
Missing	0
Mean	75.0
SD	22.44
Median	75
Q1 -- Q3	62.5 -- 87.5
Min. -- Max.	0.0 -- 100.0
AOI5	
N	136
Missing	4
Mean	58.9
SD	19.42
Median	58.3
Q1 -- Q3	41.7 -- 66.7
Min. -- Max.	16.7 -- 100.0
AOI6	
N	137
Missing	3
Mean	39.1
SD	12.85
Median	41.7
Q1 -- Q3	33.3 -- 41.7
Min. -- Max.	8.3 -- 75.0
